# Establishing a core outcome set for neurogenic bladder trials: study protocol for a scoping review and Delphi surveys

**DOI:** 10.1186/s13063-022-06419-1

**Published:** 2022-06-13

**Authors:** Yan Zhang, Yamin Chen, Mingming Niu, Yuanyuan Li, Jiaoyan Zhang, Li Zhang, Fangfang Wu, Qingyun Chen, Huijin Yu, Jinhui Tian

**Affiliations:** 1 Department of Spinal Cord Injury Rehabilitation, Gansu Province Hospital Rehabilitation Center, 53 Dingxi Road, Chengguan District, Lanzhou, 730000 Gansu China; 2 Evidence-Based Medicine Center, School of Basic Medical Sciences, Lanzhou University, No.199, Donggang West Road, Lanzhou City, 730000 Gansu Province China; 3 The Third Ward of Cardiovascular Clinical Medical Center, Affiliated Hospital of Gansu University of Chinese Medicine, Lanzhou, 730000 China; 4 School of Nursing, Shangluo Vocational and Technical College, City, Shangluo, 726000 China; 5 Department of Clinical Laboratory, The Sixth People’s Hospital of Chengdu City, Chengdu, 610000 Sichuan China; 6 School of Basic Medical Sciences, Lanzhou University, Lanzhou, 730000 Gansu China; 7 Key Laboratory of Evidence-Based Medicine and Knowledge Translation of Gansu Province, Lanzhou, 730000 Gansu China

**Keywords:** Neurogenic bladder, Core outcome set, Outcome measurement instruments, Protocol

## Abstract

**Background:**

Neurogenic bladder (NGB) is a chronic and disabling condition with a high prevalence rate, which can cause economic burden on patients and their families and reduce the quality of life of patients. Researchers have carried out a large number of clinical trials on the effectiveness and safety of different interventions for the treatment of NGB. The published clinical trials of NGB generally suffered from inconsistent and irregular reporting of outcome indicators. To facilitate future research studies of NGB, a core outcome set (COS) is required, which helps translate the results into high-quality evidence.

**Methods and analysis:**

This mixed-method project has four phases instrument: in phase 1, a scoping review of the literature to identify outcomes that have been reported in clinical trials and systematic reviews of clinical trials of interventions for NGB; in phase 2, a qualitative component using interviews to obtain the views of NGB patients, families, and their caregivers; in phase 3, Delphi survey among stakeholders to prioritize the core outcomes; and in phase 4, a face-to-face consensus meeting to discuss and agree on the final NBG COS.

**Conclusions:**

We will develop a COS that should be reported in future clinical trials of NGB.

**Trial registration:**

Core Outcome Measures in Effectiveness Trials (COMET) Initiative database registration: http://www.comet-initiative.org/studies/details/1985. Registered on 02 January 2022. INPLASY INPLASY202210007

## Introduction

Neurogenic bladder (NGB), also known as neurogenic lower urinary tract dysfunction (NLUTD), refers to describe these alterations in bladder function that are caused by neurologic dysfunction that results from disease or injury, which is one of the common complications in patients with neuropathy such as stroke, spinal cord injury, peripheral nerve injury, multiple sclerosis, Parkinson’s disease, cerebral palsy, spina bifida, and diabetes [1–3]. Common symptoms of NGB patients include urinary tract-related symptoms such as urgency, frequent urination, nocturia, urinary incontinence, labored urination, inability to empty the bladder, detrusor overactivity, and painful urination. In addition, NGB patients may experience extra-urinary tract symptoms such as bowel dysfunction, sexual dysfunction, anxiety, depression, sleep disturbance, fatigue, and pain [4–6]. A study result on the prevalence of NGB with traumatic and non-traumatic spinal cord injury in SARAH Network hospitals of Brazilians was 94.65% [7]. Spinal cord injury led to NGB in about 70–84% of patients [8]. High-quality evidence provided by a review indicated that 40 to 90% of patients with multiple sclerosis (MS), 37 to 72% of patients with Parkinson’s disease, and 15% of patients with stroke led to NGB [8]. The results of a retrospective analysis from April 1, 2002, to March 31, 2007, showed that more than one-third of NGB patients were hospitalized [3]. The total cost of the emergency department of NGB alone was estimated to be extensive, ranging from $87 to $325 million [9]. Modeled estimates of US spending on personal health care and public health showed NGB accounted for only 1% of the total health care costs of the conditions associated with its development, from 1996 through 2013 [10]. In summary, NGB is a chronic and disabling condition with a high prevalence rate, which can cause economic burden on patients and their families and reduce the quality of life of patients.

Researchers have carried out a large number of clinical trials on the effectiveness and safety of different interventions for the treatment of NGB. There are various interventions, such as rehabilitation, electrical stimulation therapy, acupuncture, drug therapy, catheterization, surgical treatment, magnetic stimulation, and traditional Chinese medicine treatment [11–13]. The complexity and variety of NGB treatment options make it more important to evaluate the efficacy of different interventions. How to choose the best clinical practice treatment measures based on high-quality evidence is a problem that needs to be solved urgently. Multiple systematic reviews or meta-analyses, due to the irregular and incomplete reporting of outcomes, cannot get reliable conclusions. At present, the published clinical trials of NGB generally suffer from inconsistent and irregular reporting of outcome indicators. The status of adverse events is generally underreported, which will cause waste of resources, mislead clinical practice, and affect clinical decision-making based on evidence-based evidence.

To facilitate future research studies of NGB, a core outcome set (COS) is required. COS, as an agreed-upon standard set of outcome measures for a particular disease or condition, can reduce the risk of reporting bias by prompting researchers to focus their reporting on a more specific set of outcomes deemed by stakeholder consensus as most appropriate to their field [14–16]. Through standardized outcome reporting in a given field, there is also the potential to reduce research waste, increase the utility of RCTs, facilitate treatment comparisons across different sources of evidence, and expedite the production of systematic reviews, meta-analyses, and evidence-based clinical guidelines [17, 18]. Our study will develop a COS for NGB clinical trials.

## Scope and aim

### Aim

The aim of our study is to develop a COS for NGB clinical trials. If necessary, we will develop a domain of COS according to the type of intervention.

### Scope

The health condition for this study is on NGB. Patients with NGB aged 18 and above will be included. This COS will cover all interventions. The COS is designed for use in both research and routine clinical care, in any health care system. We plan to involve patients, caregivers, healthcare professionals, and researchers in developing the COS in order to identify the outcomes of most importance to all stakeholders.

### Patient and public involvement

We will recruit patients and the public (caregivers and journal editors) to participate in semi-structured interviews or a questionnaire-based survey.

## Methods

### Study design

This study protocol is written with reference to the Core Outcome Set-STAndards for Reporting (COS-STAR), the Core Outcome Set-STAndards for Development (COS-STAD), and the Core Outcome Set-STAndardised Protocol Items (COS-STAP) statement [15, 16, 19]. We will use a mixed-method design involving both qualitative and quantitative methodologies to achieve consensus on the COS [20]. The COS development will follow the Core Outcome Measures in Effectiveness Trials (COMET) handbook [17]. This COS development study was registered with the COMET Initiative in December 2021 (https://www.comet-initiative.org/Studies/Details/1985). This study was also registered on INPLASY (registration number: INPLASY202210007).

**Fig. 1:**
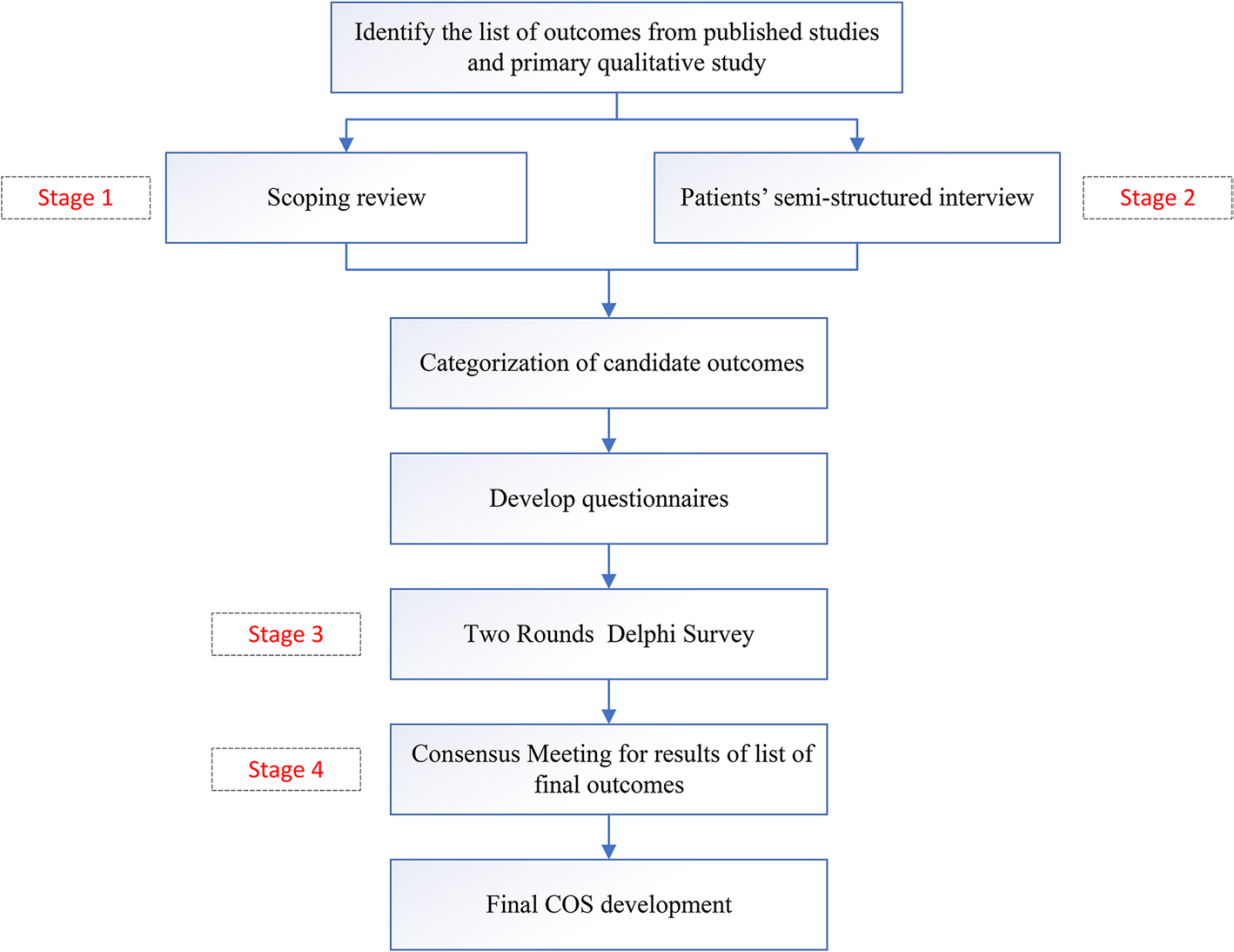
Flow chart of the study design for COS on neurogenic bladder

A multistage study is necessary to achieve this goal. The design of this study is outlined in Fig. 1 and is divided into four stages: a scoping review, stakeholder key informant interviews, an online international Delphi survey, and a consensus meeting.

Phase 1: A scoping review of the literature to identify outcomes that have been reported in clinical trials and systematic reviews of interventions for NGB

Phase 2: A qualitative component using interviews to obtain the views of NGB patients, families, and their caregivers

Phase 3: Delphi survey among stakeholders to prioritize the core outcomes

Phase 4: A consensus meeting to discuss and agree on the final NBG COS

The steering group consists of public and patient involvement representatives, journal editors, methodologists, statisticians, senior nurses, clinicians, experts in COS development, and researchers with expertise in NGB. The collective knowledge of this group will inform the development of this COS.

### Phase 1: scoping review

#### Research question: what outcomes should NGB studies report?

We will carry out a scoping review of clinical trials and systematic reviews of clinical trials evaluating the interventions of NGB to identify and collate reported outcomes. Study eligibility criteria defined by PICOS are shown in Table 1.

**Table 1:** Study eligibility criteria defined by PICOS

PICOS component	Description
Population	Adults diagnosed with NGB
Interventions/exposures	No restrictions on interventions and exposures
Comparators	No restrictions on comparators
Outcomes	No restrictions on outcomes
Study design	Non-animal intervention studies of NGB

*NGB* neurogenic bladder

#### Literature search

The following eight databases will be searched for relevant studies: Web of Science, PubMed, Embase.com, Cochrane Library, CINAHL, Wanfang database, CNKI, and Chinese BioMedical Database. ClinicalTrials.gov, International Standard Randomized Controlled Trial Number Register, Chinese Clinical Trial Registry, the World Health Organization’s International Clinical Trials Registry Platform, PROSPERO, and INPLASY will also be searched for relevant, ongoing trials. Relevant key search terms will include “neurogenic bladder,” “neurogenic urinary bladder,” “neurogenic dysfunction of the urinary bladder,” “urinary bladder neurogenic dysfunction,” “neuropathic bladder,” “bladder neurogenesis,” “neurogenic lower urinary tract dysfunction,” “neurogenic overactive bladder,” “neurogenic detrusor overactivity,” and “overactive bladder syndrome.” Randomized or non-randomized studies, prospective or retrospective studies, will be included. We will exclude cohort studies, case–control studies, case series, case reports, qualitative research, economic evaluation studies, letters to the editor, commentaries, editorials, conference abstracts that do not describe clinical outcomes, and reviews that do not report on outcomes or contain original research.

#### Study selection and data extraction

Two reviewers will independently screen the literature, extract data, and cross-check them according to the inclusion and exclusion criteria. Any disagreements will be resolved through discussion after a thorough reading of the paper or by consulting with the third researcher. An extraction table will be designed by the working group to collect relevant information on the study design, setting, demographics of participants, types of intervention, and outcomes [21]. Data extracted on the outcomes reported will include the name, definition, measurement time points, and measurement instruments or methods. Extracted outcomes will be reported verbatim to ensure authenticity and traceability from the original data. Any alteration of the data extracted during the process will be recorded.

As the aim of this scoping review is to identify all reported outcomes in order to generate a long list of outcomes to inform the development of the COS, and there is no validated tool to assess the quality of outcome reporting, it is decided that the quality of outcome reporting of included studies would not be assessed [22].

#### Data analysis and presentation

We will enter the data into Microsoft Excel in order to aid tabulation and analysis. Outcomes will be grouped under domains following a review of the outcomes. We will identify the number of trials that reported each outcome domain. For each outcome domain, we will assess the number of different outcomes (including measures) and the number of trials that assessed each specific outcome. We will perform statistical analyses of the frequency of outcomes using the software package R version 3.2.3 (R Foundation for Statistical Computing, Vienna, Austria) [23].

### Phase 2: patients’ semi-structured interview

#### The inclusion/exclusion criteria of stakeholders

It is necessary to obtain the opinion from stakeholders on NGB. Semi-structured interviews will ensure that certain topics are discussed, allowing patients the flexibility to go into details when needed. During patient interviews, information about health status, experiences and expectations regarding treatment, and outcomes relevant from the patients’ perspective will be collected. Patients ≥ 18 years of age diagnosed with NGB as well as their family members or caregivers will be invited to participate in the semi-structured interview (Table 2). Informed consent is necessary to recruit participants into this study. Patients will be reassured at the start of the interview that they can opt out at any moment during the interview should they feel unable or uncomfortable.

**Table 2:** The inclusion and exclusion criteria for semi-structured interview

Inclusion criteria	Exclusion criteria
Patients with NGB	Patients with severe mental disease, cancer, and other life-threatening diseases
Patients ≥18 years of age	
Caregivers who are taking care of patients with NGB	
Patients/caregivers who signed the informed consent forms	

*NGB* neurogenic bladder

#### Sampling

Patient interviews will be conducted in China to ensure that representative views of patients are included. We plan to approach potential patients in the inpatient ward of Rehabilitation Center Hospital of Gansu Province, the First Affiliated Hospital of Xi’an Jiaotong University, the Second Hospital of Lanzhou University, the Sixth People’s Hospital of Chengdu City, and Beijing University of Chinese Medicine. There are no robust standards for the sample size of semi-structured interviews. More than 60 patients or caregivers will be recruited following the purpose of obtaining the overall outcomes. We believe this will achieve sufficient saturation in the semi-structured interview, which means no new ideas occur [24]. They can complete the questionnaire with the help of the investigator. However, we cannot guarantee the representativeness of the sample during the sample selection. To address this issue, we will select patients varying in gender, age, disease stage, disease severity, levels of education, occupation, family income, etc. [25]. This will ensure that patients are as different as possible in the sampling process.

#### Data collection and analysis

The questions of the semi-structured interviews are as follows:

1. When was your NBG diagnosed?
2. What are the most disturbing issues for you after the NGB? Or what problems do you want to solve?
3. Which outcomes are important to you? Which one is the most important?

If there are new outcomes recommended by patients or caregivers, two researchers will identify if they are new ones. The new ones will be discussed if measurable. If so, they will be included in the Delphi Survey.

#### Categorization of candidate outcomes

Clinician-reported and patient-reported outcomes resulting from the scoping review of the literature and patient interviews will be categorized into domains using the COMET taxonomy [17]. The outcomes will be mapped by the Study Management Group to better understand the spread of outcomes but will not necessarily guide the formation of the COS. Disagreement will be resolved through discussion. Next, the outcomes will be formatted into questions and taken forward to the Delphi consensus. To ensure appropriate phrasing and understanding for all stakeholders, outcomes will be translated, and lay definitions will be provided in different languages. The surveys will be pilot tested [26].

### Phase 3: Delphi survey

#### Stakeholders’ involvement

We will invite health professionals, such as clinicians, senior nurses and researchers in NGB, and methodologists in evidence-based medicine to participate in the two rounds of the Delphi survey. The inclusion and exclusion criteria for participants in the Delphi survey are given in Table 3.

**Table 3:** The inclusion and exclusion criteria for health professionals in the Delphi survey

Inclusion criteria	Exclusion criteria
Health professionals with at least a master’s degree.	None
Health professionals who have at least 5 years of work experience.	
Clinicians and nurses who work in tertiary hospitals in China.	
Researchers who have participated in clinical trials of NGB or conducted systematic reviews of NGB in the past 10 years.	
There will be no restriction on the professionals’ geographical area.	

*NGB* neurogenic bladder

#### Sampling

There is no consensus on the optimal sample size for a Delphi study. In previous studies, the sample size of health professionals ranged from 12 to 174 [17]. In this research, we will try to invite every eligible participant from the aforementioned institutions to participate in the Delphi survey. We will encourage participants to forward the online survey to their colleagues who are eligible. The sample size for each stakeholder will be at least 100.

#### Round 1 of the Delphi survey

We will invite participants through email with links to the original questionnaire. Before carrying out the survey, all Delphi survey texts will be approved by the advisory group to confirm the readability of the language. Round 1 of the Delphi survey will last for 3 weeks. We will send emails or messages to remind potential participants to complete the Delphi survey at the end of the second weekend. A 9-point scoring system will be used in the questionnaire, wherein “1–3” indicates that the outcome is not important in the COS, “4–6” indicates that the outcome is important but not critical in the COS, and “7–9” indicates that the outcome is critical in the COS [24, 27]. Participants will also have the option to choose “unclear” for each outcome, if they find it difficult to score in terms of importance. At the end of the questionnaire, there will be one open-ended question: which outcomes do you think are important but are not included in the questionnaire [24]?

#### Data analysis for round 1 of the Delphi survey

Data analysis for round 1 of the Delphi survey will include the frequencies of the response options for each outcome. If an outcome is scored as 7–9 by no more than 10% of participants who complete the questionnaire, it will be excluded from round 2 of the Delphi survey. If participants recommend outcomes that are not included in round 1 of the Delphi survey, two researchers will identify if they are new ones. New outcomes will be included in round 2 of the Delphi survey.

#### Round 2 of the Delphi survey

Round 2 of the Delphi survey will be sent to participants who complete round 1 of the Delphi survey. In the questionnaire, the participants will receive their score from round 1 of the Delphi survey and the score distribution of their own stakeholders. They will be asked to re-score the outcomes within 3 weeks. We will send emails or messages to remind participants to complete the Delphi survey at the end of the 2nd weekend. If the response rate is < 80%, we will keep the Delphi survey open longer, or invite other eligible people to participate in the survey.

#### Data analysis for round 2 of the Delphi survey

Data analysis for round 2 of the Delphi survey will include the response rate; the frequencies of the response options for each outcome from different stakeholders; the number of participants who score differently among those who complete both round 1 and round 2; the outcomes that achieve “consensus in,” “consensus out,” and “no consensus”; and the potential attrition bias. The statistically significant outcomes will be discussed in the consensus meeting [24]. As shown in Table 4, each outcome will be defined as 3 categories [24, 28]. SPSS 25.0 will be used to calculate the positive coefficient, degree of authority, and coordination coefficient of participants in order to demonstrate the validity of the two rounds of Delphi expert consultation.

**Table 4:** Definition of consensus

Classification consensus	Criteria	Interpretation
Consensus in	≥ 70% of the participants rated the outcome 7–9 and less than 15% rated the outcome 1–3 or the average patient rating is ≥7, regardless of other scores	Outcome is important
Consensus out	≥ 70% of the participants rated the outcome 1–3 and less than 15% rated the outcome 7–9	Outcome is not important
Consensus to be further discussed	All other results	Potentially important outcome

### Phase 4: consensus meeting

#### Stakeholder selection

We will hold a face-to-face consensus meeting after analyzing the data of the surveys. We will also invite different stakeholders to participate. For the participants, the inclusion and exclusion criteria are as listed in Table 5. For the patients and the public, we will respectively invite patients, caregivers, and participant who completes the questionnaire from each stakeholder to attend the meeting, with the percentage of patients and caregivers ranging from 4 to 50% of all consensus meeting attendance.

**Table 5:** The inclusion and exclusion criteria for health professionals in the consensus meeting

Inclusion criteria	Exclusion criteria
Health professionals with at least a master’s degree.	None
Health professionals who have at least 5 years of work experience.	
Clinicians and nurses who work in tertiary hospitals in China.	
Researchers who have participated in clinical trials of NGB or conducted systematic reviews of NGB in the past 10 years.	
Journal editors should have at least 3 years’ work experience.	
Policymakers who have at least 5 years of work experience.	
There will be no restriction on the professionals’ geographical area.	

*NGB* neurogenic bladder

#### Sampling

There is no standard sample size calculation method for the process of the consensus meeting. To obtain different stakeholders’ perspectives, as well as to improve consensus achievement, we will invite at least five participants from each stakeholder to attend the consensus meeting.

#### Consensus meeting process

Participants who have participated in the two rounds of the Delphi survey will be requested to attend face-to-face meetings. Journal editors and policymakers will also be invited. The consensus meeting will be held in Lanzhou City, Gansu Province, China. It will last at least 1 day. In the consensus meeting, we will report the results of round 2 of the Delphi survey for health professionals and the results of the survey for patients and the public. The outcomes which are achieved “consensus out” by all stakeholders will be excluded. The outcomes which are achieved “consensus in” by all stakeholders will be sent to the Steering Committee and participants on the day before the consensus meeting.

In the consensus meeting, if the participants disagree with any outcome that achieved “consensus in” by all stakeholders to include in the COS, they will further discuss it. No consensus outcomes will be discussed one by one. Then, all of the participants in the consensus meeting will be anonymously asked to vote as “controversial” or “no consensus” outcomes reached. The ones which are voted by ≥ 70% participants will be included in the final COS.

### Dissemination

The finished COS will follow the recommendation of recent studies on dissemination strategies [29, 30]. Each of the participants of the COS will be asked to implement the COS in their future clinical trials of NGB treatment and recommend this COS to their colleagues and other potential researchers. We will communicate our results via peer-review publication, conference presentations, and professional societies and also via our institution’s social media platforms. We will post our final COS information on the COMET website.

## Discussion

NGB is a common problem faced by global health care institutions, which seriously threatens the life and health of patients. At present, the published clinical trials of NGB generally generally suffered from inconsistent and irregular reporting of outcome indicators. This study protocol is the first COS to be registered on the COMET website. The COS is a standardized minimum outcome set for application in clinical trials and systematic reviews, which helps translate the results into high-quality evidence. The use of COS in the synthesis and conversion of evidence is essential. We believe that it is necessary to develop a COS for NGB, which can provide the standardization of NGB clinical trial results, improving the quality of evidence for NGB.

This protocol design establishes a comprehensive review of the international and Chinese literature by conducting semi-structured interviews, Delphi surveys, and a consensus meeting to fully adopt the views of multiple stakeholder groups, which can ensure the feasibility and promotion of COS in future clinical studies. The development of COS ensures the consistency of reporting clinical study outcomes for NGB in the future, assisting in reducing reporting bias. The results of different clinical trials can be compared and merged in the future to improve the value of clinical studies and reduce the waste of study resources [31].

## Conclusion

The COS development described in this protocol will identify which outcomes should be recommended to be reported in NGB clinical trials.

## Status

At the time of submission of this manuscript, the search strategy for the rapid review has been peer-reviewed and implemented, and screening against inclusion/exclusion criteria is underway.

## Data Availability

The data presented in this study are available on request from the corresponding author.

## Abbreviations

NGB: Neurogenic bladder
NLUTD: Neurogenic lower urinary tract dysfunction
MS: Multiple sclerosis
COS: Core outcome set
COS-STAR: Core Outcome Set-STAndards for Reporting
COS-STAD: The Core Outcome Set-STAndards for Development
COS-STAP: The Core Outcome Set-STAndardised Protocol Items
COMET: The Core Outcome Measures in Effectiveness Trials
